# The predictive role of psychotic-like experiences in suicidal ideation among technical secondary school and college students during the COVID-19 pandemic

**DOI:** 10.1186/s12888-023-05025-y

**Published:** 2023-07-19

**Authors:** Meng Sun, Dongfang Wang, Ling Jing, Liang Zhou

**Affiliations:** 1 Department of Social Psychiatry, the Affiliated Brain Hospital of Guangzhou Medical University, 36 Mingxin Road, Fangcun, Liwan District, Guangzhou, 510370 China; 2 School of Psychology, South China Normal University, Guangzhou, Guangdong China; 3 Case Western Reserve University School of Medicine, Cleveland, OH USA

**Keywords:** Psychotic-like experiences, Suicide, Resilience, Social support, Students, Young adults, Fear

## Abstract

**Background:**

Previous research has shown the strong association between psychotic-like experiences (PLEs) and suicide. However, the predictive role of PLEs in suicidal ideation (SI) during the COVID-19 pandemic remains unclear.

**Aims:**

This study aimed to explore the association between PLEs before the pandemic and SI during the pandemic among late adolescents.

**Methods:**

A total of 938 technical secondary school and college students completed both waves of the online survey before and during the pandemic. PLEs were assessed through the 15-item Positive Subscale of the Community Assessment of Psychic Experiences. SI was evaluated by the frequency of SI during the pandemic.

**Results:**

In early stage of the pandemic, most students had low frequent SI, and only 3.3% students showed high frequent SI. Compared to the low frequent group, the high frequent group exhibited significantly higher levels of PLEs (*p* < 0.001) and scored lower in resilience (*p* = .001) and perceived social support (*p* = .008) across the two timepoints. PLEs were significantly associated with higher risk of high frequent SI (OR = 2.56, 95%: 1.07–6.13), while better resilience (OR: 0.93, 95% CI: 0.88–0.99) and stronger perceived social support (OR: 0.96, 95% CI: 0.93–0.99) appeared to be protective factors. No interactions were found among PLEs and other psychosocial and psychological factors.

**Conclusions:**

PLEs may increase the risk of SI in early stage of the pandemic, while good resilience and adequate social support can help weaken the risk.

**Supplementary Information:**

The online version contains supplementary material available at 10.1186/s12888-023-05025-y.

## Background

Suicide is an important public health problem which occurs throughout the lifespan. Statistically, it has been the second leading cause of death among college-aged individuals globally [1]. In China, suicide has accounted for over 40% of abnormal deaths in Chinese college students [2]. Since the outbreak of the coronavirus disease 2019 (COVID-19) at the end of 2019, concerns have been expressed that suicide rates may be increased as a result of the pandemic [3, 4], due to the stress from the pandemic itself and subsequent adverse effects caused by self-isolation, entrapment, and loneliness. For college students who have had their education interrupted due to the pandemic [5], they additionally have anxiety about their prospects, which may further lead to the deterioration of their mental health. A CDC survey has reported a significantly greater rate of suicidal ideation (SI) among college-aged adolescents compared to the general population during the pandemic (25.5% versus 10.7%) [6]. Despite the alarming rates of suicide among this population, there has been limited research addressing early screening and suicide prevention for college students [7, 8]. Therefore, it is urgently needed to detect reliable predictive indicators of suicide and to develop an effective suicide prediction model among this population. Psychotic-like experiences (PLEs), which are defined as experiences that resemble the positive symptoms of psychosis in general population [9], have been found to be strongly related to the onset of later mental disorders [10, 11]. In recent years, a growing volume of research has also suggested that individuals with PLEs have a significantly increased risk of subsequent suicidal thoughts and behaviors [12], and the persistence of SI [13]. The population-attributable fraction showed that PLEs accounted for about 10% SI and nearly a quarter of suicide attempt (SA) and suicidal death. Among late adolescents, those with PLEs even had over five times higher risk of SI [13]. Although PLEs and suicides share a battery of risk and protective factors, such as co-occurring psychopathology (e.g. depression and anxiety), psychological factors and socio-demographic factors, their association seemed to persist even in those studies where some of these factors were adjusted [12, 14]. Therefore, PLEs seem to be a promising predictor for suicide independent of the other psychopathology, and appear to be a promising but under-recognized predictor of suicide among late adolescents.

Although the strong relationship between PLEs and suicide has been revealed, many previous studies adopted a cross-sectional study design [15, 16], and thus did not provide convincing evidence for causality. Besides, several demographic characteristics (e.g. left-behind status and socioeconomic status) and psychosocial and psychological factors (e.g. childhood trauma, perceived social support and resilience) were not comprehensively considered in previous models, which may bias their association [12, 14]. Finally, as the pandemic is a chronic phenomenon with uncertain and sustained biopsychosocial effects [17], it may have some impact on the predictive role of PLEs in SI. However, it remains unclear whether PLEs can still predict SI in the context of the pandemic. In this study, we adopted a two-wave online survey among technical secondary school and college students before and after the COVID-19 pandemic. We aimed to explore the predictive role of PLEs in SI in early stage of the pandemic, as well as to assess other possible influencing factors, in order to provide useful information for suicide prevention. We also evaluated the changes of PLEs and other psychosocial and psychological factors among individuals with high frequent SI and low frequent SI in the context of the COVID-19 pandemic, which may help further understand the impact of the pandemic on mental health.

## Methods

### Participants and procedure

Two-wave online survey was conducted in a convenience sample of students from five technical secondary schools and six colleges in four provinces (Guangdong, Henan, Hunan, Zhejiang) in China. From October 2019 to November 2019 (before the pandemic), students completed the first wave of the survey by scanning the Quick Response (QR) code of the questionnaire with mobile phones in classrooms. All participants were asked to register their contact information if they were willing to participate in the subsequent survey. From April 2020 to May 2020 (in early stage of the pandemic), the second wave of the survey was conducted through sending QR to their registered mobile number or email address.

### Measures

#### Socio-demographic characteristics

Socio-demographic information collected for the first wave of the survey included: age, sex, ethnicity, family income, parental marital status, “left-behind” child status (referring to those left behind in their hometown by one or both of their migrant worker parents) [18], single child status, history of mental disorders, chronic physical conditions (having at least one of the following: arthritis, angina, asthma, diabetes, visual impairment or hearing problems) [19].

For the second wave of the survey, some COVID-19 related information was collected, including residence location (urban, town, or rural) during the COVID-19 pandemic, whether or not they were living in Hubei Province during the COVID-19 pandemic, whether or not they had been infected with COVID-19, and whether or not they had relatives or acquaintances (e.g. friends, neighbors, classmates, or family members) infected with COVID-19.

### Suicidal ideation

Suicidal ideation (SI) during the COVID-19 pandemic was evaluated by one item from the Psychological Questionnaire for Public Health Emergency (PQPHE) [20]. Response to the item “Have you ever considered to die during the pandemic” ranges from 0-seldom, 1-sometimes, 2-often, to 3-nearly always.

#### PLEs

The 15-item Positive Subscale of the Community Assessment of Psychic Experiences (CAPE-P15) was used to measure PLEs in the past month during both waves of the survey [21]. Response to each item ranges from 1-never, 2-sometimes, 3-often, to 4-nearly always. The weighted score was calculated by dividing the sum of all item scores by the number of valid items. The Chinese version of the CAPE-P15 has been found to have good reliability and validity in late adolescents [22]. Besides, a cut-off weighted score of 1.57 has been identified to detect genuine PLEs in our previous research [23]. In this sample, the CAPE-P15 also showed high internal consistency both before and during the pandemic [24].

### Other psychosocial and psychological factors

The Childhood Trauma Questionnaire (CTQ) was used to measure childhood trauma before age 16 during the first wave of the survey. Higher total scores indicate more childhood traumas experienced. The reliability and validity of the CTQ have been certified in the Chinese population [25]. In this study, Cronbach’s α for the total score was 0.81.

The 10-item Connor-Davidson Resilience Scale (CD-RISC-10) was used to assess resilience in this study. Higher total scores indicate better resilience. The Chinese version of the CD-RISC-10 has shown adequate psychometric properties [26]. In this study, the scale was used in both waves of the survey, and the Cronbach’s α for the total scores were 0.92 before the pandemic and 0.94 during the pandemic.

The Multidimensional Scale of Perceived Social Support (MSPSS) was used to measure subjective perceived social support in both waves of the survey. Higher total scores indicate higher levels of perceived social support. The psychometric properties of the MSPSS has been certified in the Chinese population [27]. In this study, the Cronbach’s α for the total scores were 0.94 before the pandemic and 0.96 during the pandemic.

### Statistical analysis

Participants who did not complete both waves of the survey were excluded for analysis. The comparisons of socio-demographic characteristics and psychosocial and psychological factors between participants who follow-up and those lost to follow-up can refer to our previous study [24, 28]. Additionally, we also excluded those with the response time of less than five minutes during each wave of the survey in order to ensure the quality of the survey responses.

First, the prevalence of different frequencies of SI was calculated. According to previous research, much more adverse effects of the pandemic were observed in the students living in Hubei Province during the pandemic and those having relatives or acquaintances infected with COVID-19. Thus, these participants were excluded from the subsequent analyses, as the small sample size of this subgroup limited further exploration and their data could have skewed the overall results. After excluding these participants, the whole sample were divided into two groups based on the frequency of SI: the low frequent SI (seldom or sometimes) and high frequent SI (often or nearly always) groups. Descriptive analyses, t-tests, and chi-square tests were carried out to describe and compare the socio-demographic characteristics, PLEs and other psychosocial and psychological factors of participants with low frequent SI and those with high frequent SI. We also explored the longitudinal differences of PLEs, resilience and perceived social support between the two groups using Generalized Estimating Equations (GEEs). The models included group, timepoint, group by timepoint interaction, CTQ scores, and all socio-demographic variables, with CAPE-P15 scores, MSPSS scores, and CD-RISC-10 scores as the dependant variable, respectively. Odds ratio (OR) and 95% confidence interval (CI) were calculated to assess the effects of group, timepoint, and their interaction.

Second, an intercept model was established initially to evaluate the school/college-level heterogeneity for distribution of low frequent and high frequent SI. As no significant heterogeneity was found (*p* = .630), we conducted a binary logistic regression to explore the association between PLEs and SI. PLEs were entered as a dichotomous variable based on the cut-off value of 1.57. And then a multivariate logistic regression was conducted to further explore the association between PLEs and SI, with all socio-demographic variables, CTQ total scores, and MSPSS and CD-RISC-10 baseline total scores included in the model. We calculated ORs and 95% CI to indicate the strength of the associations between SI and all related influencing factors.

Finally, we used additive models to test interactions among PLEs, childhood trauma, resilience, and perceived social support. Compared with multiplicative interaction, additive interaction has been found to better reflect the degree of biological interaction between risk factors [29], and to be more associated with disease prevention [30]. In this study, synergy index and 95% CIs were used to evaluate the interactions based on the method described by Andersson et al. [31]. Both the calculating formula and code can be found in the above study. It was considered to be no significant interaction if the 95% CI of synergy index included 1. To simplify interpretation of the results, all continuous variables were dichotomized. Childhood trauma, resilience and perceived social support were all categorized into two groups based on their medians among this sample.

All analyses were conducted using SPSS 19.0. A two-sided *p*-value < 0.05 was considered statistically significant.

## Results

### Description of the sample

A total of 2,265 students participated in the first wave of the survey, and 938 of them were followed up in the second wave of the survey. All response times for each wave of the survey were above five minutes for these participants. Seventy-eight students refused to register their contact information at baseline. Compared to those who have registered their contact information, these students were slightly younger (*p* < 0.001), a lower proportion were female (*p* < 0.001) or left-behind children (p = .008), and more of them were single child (*p* = .010). No significant differences were found in other demographic characteristics (all *p* > .05, see Table A1).

Among all 938 participants who followed up, twenty students lived in Hubei province, no students reported having been infected with COVID-19, and eight students reported having relatives or friends infected with COVID-19.

**Fig. 1:**
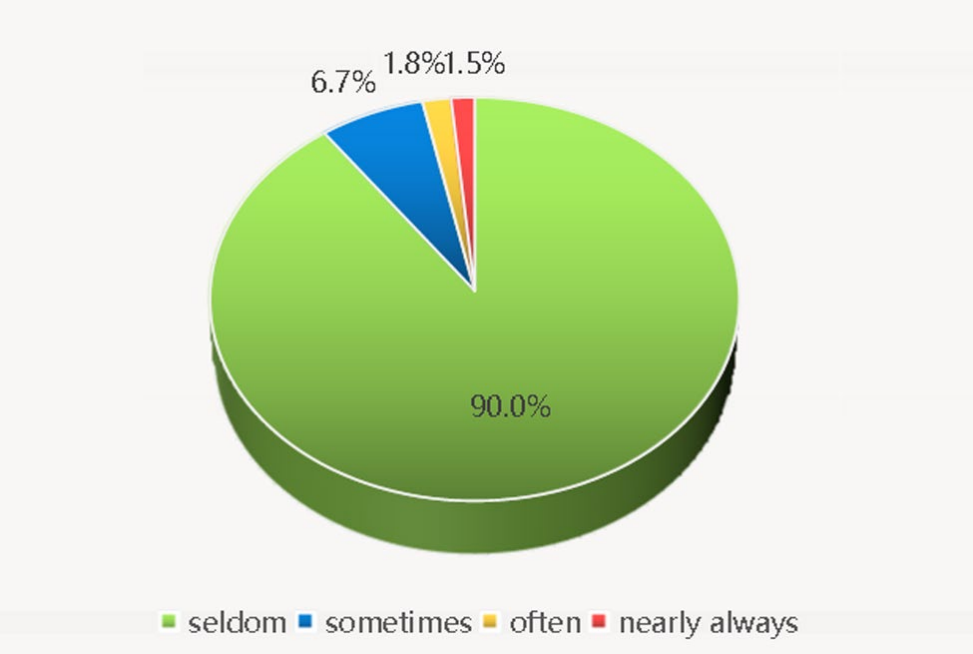
Prevalence of different frequencies of suicidal ideation during the COVID-19 pandemic (*n* = 938)

The prevalence rates of different frequencies of SI are presented in Fig. 1. Most students (*n* = 907) had low frequent SI, and only around 3% of them (*n* = 31) reported high frequent SI during the COVID-19 pandemic. Among those with high frequent SI, there were one student living in Hubei province, and one having friends infected with COVID-19. After excluding participants living in Hubei Province and those having relatives or acquaintances infected with COVID-19, 910 participants were included for the subsequent analyses.

### Comparisons of characteristics between participants with different frequencies of SI

The socio-demographic characteristics of the low frequent and high frequent SI groups are summarized in Table 1. No significant difference was observed in all socio-demographic characteristics (all *p* > .05), except for chronic physical condition. There was a significant higher prevalence rate of chronic physical illness in participants with frequent SI than in those with low frequent SI (24.1% vs. 10.7%, *p* = .033).

**Table 1:** Comparisons of characteristics between the high frequent and low frequent suicidal ideation groups

	High frequent suicidal ideation (N = 881)		Low frequent suicidal ideation (N = 29)		P-value
	Mean	SE	Mean	SE	
Age, year	17.5	0.05	17.7	0.30	0.541
	N	%	N	%	
Sex (Female)	17	69.9	616	58.6	0.219
Ethnicity (Han^a^)	25	86.2	792	89.9	0.343
Residence location					
Urban	5	17.2	108	12.3	0.137
Town	6	20.7	171	19.4	
Rural	18	62.1	602	68.3	
Family income (RMB per month)					
< 1000	2	6.9	34	3.9	0.540
1000–3000	8	27.6	280	31.8	
3000–5000	12	41.4	299	33.9	
5000–10,000	7	24.1	212	24.1	
> 10,000	0	0	56	6.4	
Parental marital status (Not current married^b^)	6	20.7	104	11.8	0.149
“Left-behind” child status (Yes)	16	55.2	422	47.9	0.457
Single child status (Yes)	7	24.1	170	19.3	0.480
History of mental disorders (Yes)	1	3.4	9	1.0	0.278
Chronic physical illness^c^ (Yes)	7	24.1	94	10.7	0.033

^a^ Han is the major ethnic group in China

^b^ Not current married included separated, divorced, and widowed

^c^ Chronic physical conditions referred to having at least one of arthritis, angina, asthma, diabetes, visual impairment, or hearing problems

**Fig. 2:**
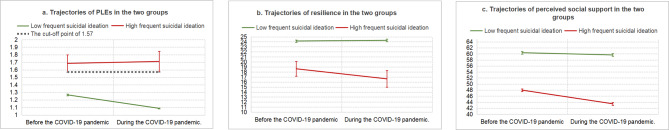
Changes of PLEs, resilience and perceived social support in the low frequent and high frequent suicidal ideation groups before and during the COVID-19 pandemic PLEs, psychotic-like experiences

As for psychological and psychosocial facors, students with high frequent SI scored significantly higher on the CTQ than those with low frequent SI (45.03 ± 2.46 vs. 38.20 ± 0.37, *p* = .001). Besides, the high frequent group also had significantly higher CAPE-P15 scores, and lower MSPSS and CD-RISC-10 scores both before and during the COVID-19 pandemic (all *p* < 0.01, see Table A2). Figure 2 shows the changes of PLEs, resilience, and perceived social support in the two groups before and during the COVID-19 pandemic. The average CAPE-P15 scores of both waves of the survey were above the cut-off value of 1.57 in the high frequent group, while the low frequent group showed both average scores below this point. Besides, from this figure we could see a flat PLEs trajectory with high scores at both waves, and a gradual decline in resilience and perceived social support from relatively low baseline scores among participants with high frequent SI, and the opposite trends among those with low frequent SI.

In the GEEs, significant group differences were found in CAPE-P15 scores (*p* < 0.001), CD-RISC-10 scores (*p* = .001), and MSPSS scores (*p* = .008), while no significant effects were found for timepoint or group by timepoint interaction (all *p* > .05), except for the main effect of timepoint on CAPE-P15 total mean scores (*p* < 0.001) (see Table 2).

**Table 2:** Longitudinal differences of PLEs, resilience, and perceived social support between groups with different frequencies of suicidal ideation

	CAPE-P15 total mean score		CD-RISC-10 total score		MSPSS total score
	OR (95%CI)		OR (95%CI)		OR (95%CI)
Group (Ref: low frequency)	1.42 (1.17, 1.72)		0.01 (0.00, 0.15)		0.00 (0.00, 0.11)
Timepoint (Ref: baseline)	0.84 (0.82, 0.85)		1.14 (0.73, 1.78)		0.48 (0.22, 1.06)
Timepoint × Group	1.23 (0.99, 1.51)		0.12 (0.00, 4.23)		0.02 (0.00, 31.45)

CAPE-P15, The 15-item positive subscale of the community assessment of psychic experiences; MSPSS, the Multidimensional Scale of Perceived Social Support; CD-RISC-10, the 10-item Connor-davidson Resilience Scale

^a^ Adjusting for all socio-demographic characteristics and childhood trauma scores

### Associations between PLEs and SI

In the unadjusted binary logistic model, participants with PLEs before the pandemic were more than four times likely to have high frequent SI compared to those without PLEs (OR = 4.65, 95% CI = 2.19–9.90). After adjusted for all socio-demographic characteristics, and other psychosocial and psychological factors, the association between PLEs and SI attenuated but persisted (OR = 2.56, 95% CI = 1.07–6.13) (Table 3). Meanwhile, higher CD-RISC-10 baseline scores (OR = 0.93, 95% CI = 0.88–0.99) as well as higher MSPSS baseline scores (OR = 0.96, 95% CI = 0.93–0.99) exhibited to be protective factors of high frequent SI.

**Table 3:** Multivariate logistic regression model of suicidal ideation in early stage of the COVID-19 pandemic (N = 910)^a^

	OR	95% CI	Wald	*p*-value
Age	0.98	0.74, 1.29	0.025	0.875
Sex (Female)	0.57	0.24, 1.34	1.689	0.194
Ethnicity (Han^a^)	0.49	0.15, 1.67	1.294	0.255
Residence location				
Urban	1.00	-	-	-
Town	0.62	0.23, 1.71	0.856	0.355
Rural	0.41	0.13, 1.28	2.371	0.124
Family income	1.00	0.66, 1.51	<0.001	0.995
Parental marital status (Not current married^b^)	1.66	0.57, 4.86	0.864	0.353
“Left-behind” child status (Yes)	1.18	0.52, 2.70	0.156	0.693
Single child status (Yes)	1.10	0.41, 2.94	0.032	0.858
History of mental disorders (Yes)	1.106	0.10, 12.17	0.007	0.934
Chronic physical illness^c^ (Yes)	1.86	0.67, 5.17	1.394	0.238
PLEs (Yes)	2.56	1.07, 6.13	4.458	0.035
CTQ, total score	0.99	0.96, 1.03	0.155	0.694
CD-RISC-10, total score	0.93	0.88, 0.99	4.575	0.032
MSPSS, total score	0.96	0.93, 0.99	5.966	0.015

PLEs, psychotic-like experiences; MSPSS, the Multidimensional Scale of Perceived Social Support; CTQ, the Childhood Trauma Questionnaire; CD-RISC-10, the 10-item Connor-davidson Resilience Scale

^a^ Han is the major ethnic group in China

^b^ Not current married included separated, divorced, and widowed

^c^ Chronic physical conditions referred to having at least one of arthritis, angina, asthma, diabetes, visual impairment, or hearing problems

### Interactions among PLEs, chilhood trauma, resilience, and perceived social support

Controlling for all socio-demographic characteristics and other psychosocial and psychological factors, we further explored the interactions among PLEs, childhood trauma, resilience, and perceived social support, with no significant interactions observed (see Table 4).

**Table 4:** Interaction of PLEs, more childhood trauma, lower perceived social support and poorer resilience in suicidal ideation during the COVID-19 pandemic

	High frequent suicidal ideation vs. Low frequent suicidal ideation	
	Synergy Index	95% CI
PLEs× more childhood trauma	1.27	0.19, 8.53
PLEs× lower perceived social support	3.51	0.02, 620.86
PLEs × poorer resilience	3.96	0.48, 33.02

PLEs, psychotic-like experiences; SI, suicidal ideation

^a^ Adjusting for all socio-demographic characteristics and psychosocial and psychological factors except the two variables tested for interaction

## Discussion

To our knowledge, this is the first study to evaluate the predictive role of PLEs in SI in the context of the COVID-19 pandemic. We also found several protective factors and examined the interactions among PLEs and other psychosocial and psychological factors. Additionally, in this study, the changes of PLEs, resilience, and perceived social support before and during the pandemic were explored among students with different frequencies of SI.

In this sample, one in ten students experienced SI in early stage of the COVID-19 pandemic. The prevalence rate is similar to that in previous research among college students (13.4%) [8] but much lower than the data from a CDC survey [6]. This may be contributed by the differences in sampling time. Data has displayed no rise in suicide rates in the early months of the pandemic compared with the expected levels based on the pre-pandemic period [32]. However, the risk of suicide related to the pandemic seems to be dynamic, and a rise has been reported following an initial decline in Japan [33].

In this study, PLEs before the pandemic was found to be associated with around 2.5 times higher risk of high frequent SI during the COVID-19 pandemic. Even after controlling for all social-demographic, psychological and psychosocial factors, PLEs appeared to contribute additional risk to high frequent SI. These results confirm the predictive effect of PLEs in SI in early stage of the pandemic, and suggest the existence of other possible mechanism underlying their association, especially during the COVID-19. In our previous research, we have found the potential moderating effects of the COVID-19 related fear in the relationship between PLEs and SI [34]. A recent study conducted in psychiatric Emergency Department further demonstrated that the COVID-19 related fear prevailed in youth in lockdown, which suggested more personalized prevention strategies targeted at this population [35].

In the current study, the unadjusted OR value in this study is comparable to the data in previous research, but much lower than that (10.01) of SA [36]. These results point toward the following hypothesis: PLEs may have more obvious advantage in prediction of suicidal behaviors (SB) than in that of suicidal thoughts [37]. Therefore, PLEs are expected to be widely used in early identification of suicide in late adolescents. However, further exploration is needed in the association between PLEs and suicide behaviors during the pandemic, as well as its potential mechanism.

We also explored the change of PLEs in the high frequent and low frequent SI groups. Different changing trends emerged in the two groups——while stable high levels of PLEs were found in the high frequent group, the low frequent group showed gradually declining PLEs. The results coincide with our previous research on the associations of PLEs and other pandemic related psychological symptoms [28]. According to the proneness-persistence-impairment model raised by van Os [38], transitory PLEs may become abnormally persistent and subsequently clinically relevant under the influence of environmental risk factors. Although the model is initially proposed for the continuum of psychosis, it also seems to apply to the associations between PLEs and other mental disorders, and suggests more focus are needed on the clinical significance of persistent PLEs.

Apart from PLEs, we also found two other influencing factors significantly related to high frequent SI. Consistent with previous research [39, 40], better resilience and higher levels of perceived social support appeared to be protective factors of high frequent SI. Additionally, trajectories of resilience and perceived social support in the high and low frequent groups are opposite to those of PLEs before and during the he COVID-19 pandemic. Students with high frequent SI showed no significant downtrend in resilience and perceived social support from relatively low baseline scores, while those with low frequent SI had stable high levels of resilience and perceived social support. It can be speculated that the pandemic and the consequent lockdown may exert more negative impact on those with low social support and poor resilience at baseline through adverse life events such as domestic violence and maltreatment [4, 41, 42], and thereby aggravating their SI during the pandemic.

We further explored additive interactions among PLEs, childhood trauma, resilience, and perceived social support on SI. Although previous studies have suggested that the association between PLEs and suicide may be moderated by some psychological factors [12, 14], no such moderation was found in this study, suggesting that childhood trauma, resilience, and perceived social support may act on distinct pathways from PLEs. However, these results need to be interpreted with caution in this relatively small sample, and further validation is needed in large sample.

There exist several strengths of this study. First, we verified the predictive effects of PLEs in SI during the pandemic for the first time with a follow-up study design, which provides new evidence for the predictive value of PLEs. Second, this study was conducted among college students, and indicated potential targets for mental health intervention in this vulnerable population. Last but not least, we confirmed distinct protective roles of resilience and social support through additive interaction models, which help better understand multiple psychological pathways underlying SI.

One of the limitations of this study is the small sample size. However, as an exploratory research, it could still suggest the predictive role of PLEs in SI during the pandemic. Small sample size generated by high attrition rate in this study can be attributed to the irresistible factors caused by the pandemic. Although we contacted all students who had registered contact information through text messages or emails, only fewer than half of these students completed the second wave of the survey. Additionally, single item was used for assessing SI. Although self-report single-item assessment was adopted in most previous research, it may have unsatisfactory precision and result in the misclassification [43]. Additionally, emotional symptoms (e.g. depression and anxiety) were not evaluated at baseline, which may lead to overestimation of the predictive effect of PLEs and weaken the power of the prediction model. Although history of mental disorders was included in the multivariate model, which can eliminate the influence to some extent, diagnosis is not equivalent to symptoms. Therefore, whether PLEs, in the absence of emotional symptoms, can predict SI remains unclear in the current study.

## Conclusions

Overall, PLEs before the pandemic seem to increase the risk for SI during the COVID-19 pandemic, and show promise as a predictive indicator for suicide prevention. Meanwhile, strong resilience and sufficient social support play a protective role in subsequent SI, and can be used as targets for suicide intervention. Future research is needed to explore the prediction of PLEs on SB, as well as the potential mechanism underlying their associations.

## Electronic supplementary material

Below is the link to the electronic supplementary material.

Supplementary Material 1: Appendix_tables (Table A1 and Table A2)

## Data Availability

The data that support the findings of this study are available on request from the corresponding author, [Zhou L].

## Abbreviations

PLEs: Psychotic-like experiences
SI: Suicidal ideation
SA: Suicide attempt
SB: Suicidal behaviors
PQPHE: The Psychological Questionnaire for Public Health Emergency
CAPE-P15: The 15-item Positive Subscale of the Community Assessment of Psychic Experiences
CTQ: The Childhood Trauma Questionnaire
CD-RISC-10: The 10-item Connor-Davidson Resilience Scale
MSPSS: The Multidimensional Scale of Perceived Social Support
GEEs: Generalized Estimating Equations
OR: Odds ratio
CI: Confidence interval
