# Supplementary material for: The predictive role of psychotic-like experiences in suicidal ideation among technical secondary school and college students during the COVID-19 pandemic
Source: BMC Psychiatry. 2023 Jul 19;23:521. doi: 10.1186/s12888-023-05025-y (PMC10357784; doi:10.1186/s12888-023-05025-y)
Supplement: Supplementary file 1 — Supplementary Material 1: Appendix_tables (Table A1 and Table A2) [file 12888_2023_5025_MOESM1_ESM.doc]

**Table A1.** Comparisons of characteristics between students who have registered their contact information and those who have not.

|  | Registered  (*n* = 2171) | | Not registered  (*n* = 78) | | *p*-value |
| --- | --- | --- | --- | --- | --- |
|  | Mean | SE | Mean | SE |
| Age, year | 17.8 | 0.04 | 16.8 | 0.20 | ＜ .001 |
|  | N | % | N | % |  |
| Sex (Female) | 1446 | 66.1 | 23 | 29.1 | ＜ .001 |
| Ethnicity (Han^a^) | 2018 | 92.3 | 77 | 97.5 | .123 |
| Residence location |  |  |  |  |  |
| Urban | 638 | 29.2 | 27 | 34.2 | .427 |
| Town | 573 | 26.2 | 16 | 20.3 |
| Rural | 975 | 44.6 | 36 | 45.6 |
| Family income (RMB per month) |  |  |  |  |  |
| <1000 | 76 | 3.5 | 5 | 6.3 | .520 |
| 1000-3000 | 590 | 27.0 | 18 | 22.8 |
| 3000-5000 | 743 | 34.0 | 24 | 30.4 |
| 5000-10000 | 595 | 27.2 | 23 | 29.1 |
| >10000 | 182 | 8.3 | 9 | 11.4 |
| Parental marital status (Not current married ^b^) | 284 | 13.0 | 6 | 7.6 | .227 |
| “Left-behind” child status (Yes) | 1027 | 47.0 | 25 | 31.6 | .008 |
| Single child status (Yes) | 439 | 20.1 | 26 | 32.9 | .010 |
| History of mental disorders (Yes) | 28 | 1.3 | 2 | 2.5 | .282 |
| Chronic physical illness ^c^  (Yes) | 293 | 13.4 | 10 | 12.7 | 1.000 |

^a^ Han is the major ethnic group in China.

^b^ Not current married included separated, divorced, and widowed.

^c^ Chronic physical conditions referred to having at least one of arthritis, angina, asthma, diabetes, visual impairment, or hearing problems

**Table A2.** Comparisons of PLEs, resilience, and perceived social support between participants with high frequent suicidal ideation and those with low frequent suicidal ideation before and during the COVID-19 pandemic.

|  | High frequent suicidal ideation  (*n* = 881) | | Low frequent suicidal ideation  (*n* = 29) | | *p*-value |
| --- | --- | --- | --- | --- | --- |
|  | Mean | SE | Mean | SE |
| CAPE-P15, total mean score |  |  |  |  |  |
| Before the pandemic | 1.69 | 0.11 | 1.27 | 0.01 | .001 |
| During the pandemic | 1.71 | 0.13 | 1.09 | 0.01 | ＜ .001 |
| CD-RISC-10, total score |  |  |  |  |  |
| Before the pandemic | 18.62 | 1.48 | 24.16 | 0.23 | ＜ .001 |
| During the pandemic | 16.62 | 1.66 | 24.29 | 0.26 | .001 |
| MSPSS, total score |  |  |  |  |  |
| Before the pandemic | 47.97 | 3.54 | 60.38 | 0.42 | .002 |
| During the pandemic | 43.45 | 2.86 | 59.64 | 0.45 | ＜ 0.001 |

CAPE-P15, The 15-item positive subscale of the community assessment of psychic experiences; MSPSS, the Multidimensional Scale of Perceived Social Support; CD-RISC-10, the 10-item Connor-davidson Resilience Scale.
